# Chronic kidney disease-related sarcopenia as a prognostic indicator in elderly haemodialysis patients

**DOI:** 10.1186/s12882-023-03175-5

**Published:** 2023-05-19

**Authors:** Madeleine Elder, Avalon Moonen, Sjorjina Crowther, Jasna Aleksova, Jacqueline Center, Grahame J. Elder

**Affiliations:** 1 School of Medicine, The University of Notre Dame Australia, Darlinghurst, NSW Australia; 2 University of Sydney, Sydney, NSW Australia; 3 Hudson Institute of Medical Research, Clayton, Vic Australia; 4 Department of Endocrinology, Monash Health, Clayton, Vic Australia; 5 Monash University, Clayton Vic, Australia; 6 Skeletal Biology Program, Garvan Institute of Medical Research, Darlinghurst, NSW Australia

**Keywords:** Sarcopenia, Chronic kidney disease, Haemodialysis, Mortality, Bioimpedance analysis

## Abstract

**Background:**

The mortality of dialysis patients greatly exceeds that of the general population and identifying predictive factors for mortality may provide opportunities for earlier intervention. This study assessed the influence of sarcopenia on mortality in patients on haemodialysis.

**Methods:**

This prospective, observational study enrolled 77 haemodialysis patients aged 60 years and over, of whom 33 (43%) were female, from two community dialysis centres. Baseline demographic and laboratory data were collected, and sarcopenia was diagnosed using grip strength, muscle mass by bioimpedance analysis (BIA) and muscle function by timed up-and-go according to European Working Group on Sarcopenia in Older People criteria. Nutritional status was assessed using a subjective nutritional assessment score, comprising functional changes in weight, appetite, gastrointestinal symptoms and energy.. A comorbidity score (maximum 7 points) was derived from the presence or absence of hypertension, ischaemic heart disease, vascular disease (cerebrovascular disease, peripheral vascular disease, and abdominal aortic aneurysm), diabetes mellitus, respiratory disease, a history of malignancy and psychiatric disease. Outcomes over six years were linked to the Australian and New Zealand Dialysis and Transplant Registry.

**Results:**

The median participant age was 71 years (range 60–87). Probable and confirmed sarcopenia was present in 55.9% and severe sarcopenia with reduced functional testing in 11.7%. Over 6 years, overall mortality was 50 of the 77 patients (65%), principally from cardiovascular events, dialysis withdrawal and infection. There were no significant survival differences between patients with no, probable, confirmed, or severe sarcopenia, or between tertiles of the nutritional assessment score. After adjustment for age, dialysis vintage, mean arterial pressure (MAP) and the total comorbidity score, no sarcopenia category predicted mortality. However, the total comorbidity score [Hazard Ratio (HR) 1.27, Confidence Intervals (CI) 1.02, 1.58, p = 0.03] and MAP (HR 0.96, CI 0.94, 0.99, P = < 0.01) predicted mortality.

**Conclusion:**

Sarcopenia is highly prevalent in elderly haemodialysis patients but is not an independent predictor of mortality. Haemodialysis patients have multiple competing risks for mortality which, in this study, was predicted by a lower MAP and a higher total comorbidity score.

**Trial registration:**

Recruitment commenced December 2011. The study was registered 10.01.2012 with the Australian New Zealand Clinical Trials Registry (ACTRN12612000048886).

**Supplementary Information:**

The online version contains supplementary material available at 10.1186/s12882-023-03175-5.

## Introduction

Chronic kidney disease (CKD) requiring renal replacement therapy is increasing in prevalence due to population ageing and escalating rates of obesity, hypertension and diabetes mellitus (DM) [1]. In Australia, people aged 65 years and over make up approximately half of the prevalent dialysis population, [2] however the median survival of dialysis patients aged 75–84 is only 3.6 years, and 2.4 years for those aged 85 and older, with most deaths due to cardiovascular events and dialysis withdrawal [2]. Dialysis is not only a costly therapy but may reduce quality of life without prolonging survival [3]. These factors emphasize the importance of assessing the utility versus futility of initiating dialysis for elderly patients with multiple comorbidities.

In 2007 the concept of protein energy wasting (PEW) was proposed by the International Society of Renal Nutrition and Metabolism (ISRNM), [4] describing the nutritional and metabolic derangements, together with low-grade inflammation, that contribute to a loss of body protein, energy stores and muscle and fat mass in patients with ESKD. The diagnostic criteria include three or more of weakness, slow gait speed, exhaustion, low physical activity, unintentional weight loss and muscle mass below the tenth percentile of an age and gender matched population [5]. Like PEW, sarcopenia results from an interaction of genetic, mechanical, hormonal, inflammatory and nutritional factors. The widely used 2019 European Working Group on Sarcopenia in Older People (EWGSOP2) definition focuses on sarcopenia as a muscle disease with low muscle strength (muscle failure), low muscle quantity (muscle mass) or quality used to confirm the diagnosis, and tests of physical performance used to identify severity [6]. In the general population, sarcopenia is closely associated with ageing, reduced quality of life and increased mortality. However, loss of muscle strength and mass occur commonly in disease states, and for patients on dialysis, sarcopenia develops as a confluence of ageing, multiple comorbidities contributing to and resulting from end stage kidney disease (ESKD), the catabolic uraemic state, dietary restrictions and reduced functional capacity. Amongst patients on dialysis, components of sarcopenia have been individually associated with mortality in meta-analysis, [7] and some studies of patients on dialysis have shown associations of sarcopenia (defined as low muscle mass plus low muscle strength or performance) with increased mortality, after adjustment for covariates [8–11]. However, this has not been consistent across all studies [12, 13] or across the categories of sarcopenia severity, after adjustment for factors such as diabetes mellitus and prevalent cardiovascular pathology [8]. Meta-analysis of these studies has been limited by variations in testing methods [7], and inclusion of younger patients may limit extrapolation of outcomes to more elderly patients commencing dialysis. Additionally, older dialysis patients are highly selected; they have achieved older age despite chronic kidney disease, and their acceptance onto dialysis indicates they are considered likely to survive for a meaningful period with dialysis support.

The aim of this study was to evaluate the prevalence of CKD-related sarcopenia in elderly, stable, community dwelling haemodialysis patients, and whether the presence of sarcopenia categorised by EWGSOP2 criteria predicted mortality. Secondary endpoints were to assess associations of mortality with a 7-point comorbidity score, a modification of the subjective global analysis (SGA) score, and with baseline laboratory and demographic data.

## Materials and methods

Patients aged 60 years and over on satellite haemodialysis within Western Sydney Local Health District were recruited in December 2011 and January 2012. All patients were dialysing 4 to 5.5 h, three-times weekly. Exclusion criteria included inability to provide informed consent, haemodialysis duration < 3 months, planned surgery (except for dialysis access), active malignancy, and conditions precluding bioimpedance analysis (BIA) or functional testing, including a cardiac pacemaker, defibrillator, or inability to ambulate.

### Data collection

Baseline demographic and clinical data were collected from patient medical records, patient interviews and by linkage to the Australia and New Zealand Dialysis and Transplant (ANZDATA) Registry. A comorbidity score (maximum 7 points) was derived from the presence or absence of hypertension, ischaemic heart disease, vascular disease (cerebrovascular disease, peripheral vascular disease, and abdominal aortic aneurysm), diabetes mellitus (DM), respiratory disease, a history of malignancy and psychiatric disease, with each scored as ‘one’ if present. Baseline laboratory values were accessed from routine monthly pre-dialysis blood samples.

Follow-up data on cessation of dialysis due to transplantation, cardiovascular events, cardiovascular mortality, withdrawal from dialysis, and all-cause mortality were obtained from the ANZDATA registry and patient medical records. Cardiovascular mortality included myocardial ischaemia and infarction, pulmonary oedema, cardiac failure and cardiac arrest, and vascular causes included pulmonary embolus, cerebrovascular accident, aortic aneurysm rupture, haemorrhage from other sites and bowel infarction, amongst other causes.

### Sarcopenia assessment

Muscle strength, body composition, functional assessment and nutritional data were all collected during a single 30-minute period following a mid-week haemodialysis session, when patients had achieved their predicted dry weight. Patients were given careful instructions according to standard protocols prior to each test, and were able to familiarise themselves with the equipment used.

1. *Muscle strength* was assessed by grip strength, knee strength and the recurrent chair stand (RCS) test, using validated techniques and standard protocols. Grip strength was measured using a Jamar dynamometer (Sammons Preston; Chicago, USA), [6] and was low if < 27 kg force (KgF) was registered for men, and < 16 KgF for women. Knee extension strength was measured using a Chatillon hand-held dynamometer (Ametek; Largo, Florida, USA) [14] and was low if < 29.5 KgF was registered for men and < 18.25 KgF for women [15]. Strength was tested three times on each arm and leg, and the maximal effort was recorded. The RCS test was assessed as the time taken for five repetitions of sit to stand from a straight-backed armless chair placed against a wall to prevent slipping and was abnormal if > 15 s.
2. *Muscle quantity* (body composition) was assessed using a bioimpedance spectrometer (Fresenius Medical Care Body Composition Monitor, Bad Homburg, Germany) and three compartment (3 C) model to quantify lean tissue mass (LTM), fat tissue mass and body fluid volume. LTM (kg) was corrected for height to give a lean tissue index (LTI) (kg/m^2^) [16, 17]. Using a standard protocol from Fresenius Healthcare, patients were positioned supine with heavy clothing removed, feet separated and arms more than 15 cm from the body. On the side opposite the fistula, distal and proximal hand electrode pads, and distal and proximal foot electrode pads were placed on skin cleaned with an alcohol swab, and electrode clamps were then attached. The test was not commenced until the patient had been supine at least 4 min, and the patient was instructed not to move or speak during the test. Height, weight, age and blood pressure were entered. Appropriate reference ranges for the LTI were based on Fresenius generated data [18], and values ≥ 2 SD from the healthy, gender-specific reference range were used to define sarcopenic cut points.
3. *Physical performance* was measured by the timed up-and-go (TUG), in which participants rise from a standard chair, walk to a point 3 m away, turn around, walk back, and sit down, and the 6-minute walk test (6MWT), in which participants walk back and forth over a level course of 10 m as quickly as they can for 6 min. An abnormal TUG was ≥ 20 s and 6MWT ≤ 400 m. All functional testing was overseen by a qualified physiotherapist.
  For analysis of the dataset, sarcopenia was defined using the 2019 EWGSOP2 criteria of muscle strength, using grip strength, mass based on BIA analysis, and function based on the TUG test, and stratified as ‘probable’, defined by low muscle strength, ‘confirmed’ defined by low strength and muscle quantity, and ‘severe’ defined by the addition of reduced physical performance [6].
4. *Nutritional status* was evaluated by a renal dietician, using a subjective scoring system described below. This ‘nutritional assessment score’ was adaptated from the 3-point Subjective Global Assessment (SGA) described by Detsky et al. [19]. Patients completed a structured evaluation form within 30 min of a dialysis session. The questionnaire asked them to rate their current appetite (excellent, good, fair or poor) and to comment on changes in appetite within the previous 2 weeks and 6 months (no change, increased or decreased). They were provided with options to rate their current food intake, to indicate whether they had symptoms of anorexia, nausea, vomiting or diarrhoea, and if so, their frequency, to rate their energy and activity levels in the last 6 months, and to provide an overall wellbeing assessment. Changes in weight in the previous 2 weeks and 6 months were assessed from dialysis records. The ratings for changes in weight, appetite, gastrointestinal symptoms and energy were scored by 2 researchers, and the scores were summed to stratify patients into one of three categories. A: well nourished, B: mildly or moderately malnourished, C: severely malnourished. While the SGA includes assessment of comorbidities, functional capacity, subcutaneous fat and muscle wasting, in this study, functional capacity, comorbidities and body composition were determined by alternate methods. They were therefore excluded from the nutritional assessment score used in this study.

### Outcomes

Follow-up was to 31st December 2017, six years from recruitment. The primary endpoint was all-cause mortality related to the absence or presence of sarcopenia by category; presumed, confirmed or severe. Secondary endpoints were associations of mortality with the baseline 7-point comorbidity score, the modified SGA, and baseline laboratory and demographic indices.

### Statistical analysis

Independent t-tests and Spearman’s Rank-Order correlation were used to assess associations between baseline ordinal and continuous variables, and Chi-square tests were used for categorical variables. Cox proportional hazards regression models were used to assess the associations of baseline variables to all-cause mortality, and to cardiovascular mortality. Values are expressed as mean ± SD, median (range) or percentage as appropriate, and *p-*values < 0.05 were considered statistically significant. The study comprised a convenience sample of all patients within two satellite dialysis units who met inclusion criteria, were willing to participate and were able to consent between December 2011 and January 2012. Data was analysed using SPSS 27.0 (Armonk, NY: IBM Corp).

### Ethics

All patients provided informed consent and the study was performed in accordance with the Declaration of Helsinki. The study was approved by The University of Notre Dame Australia Human Research Ethics Committee (012035 S) and the Western Sydney Local Health District Human Research Ethics Committee (2011/11/6.2 (3423)) on the 17/11/2011 and was registered with the Australian New Zealand Clinical Trials Registry (ACTRN12612000048886) on 10th January 2012.

## Results

**Fig. 1:**
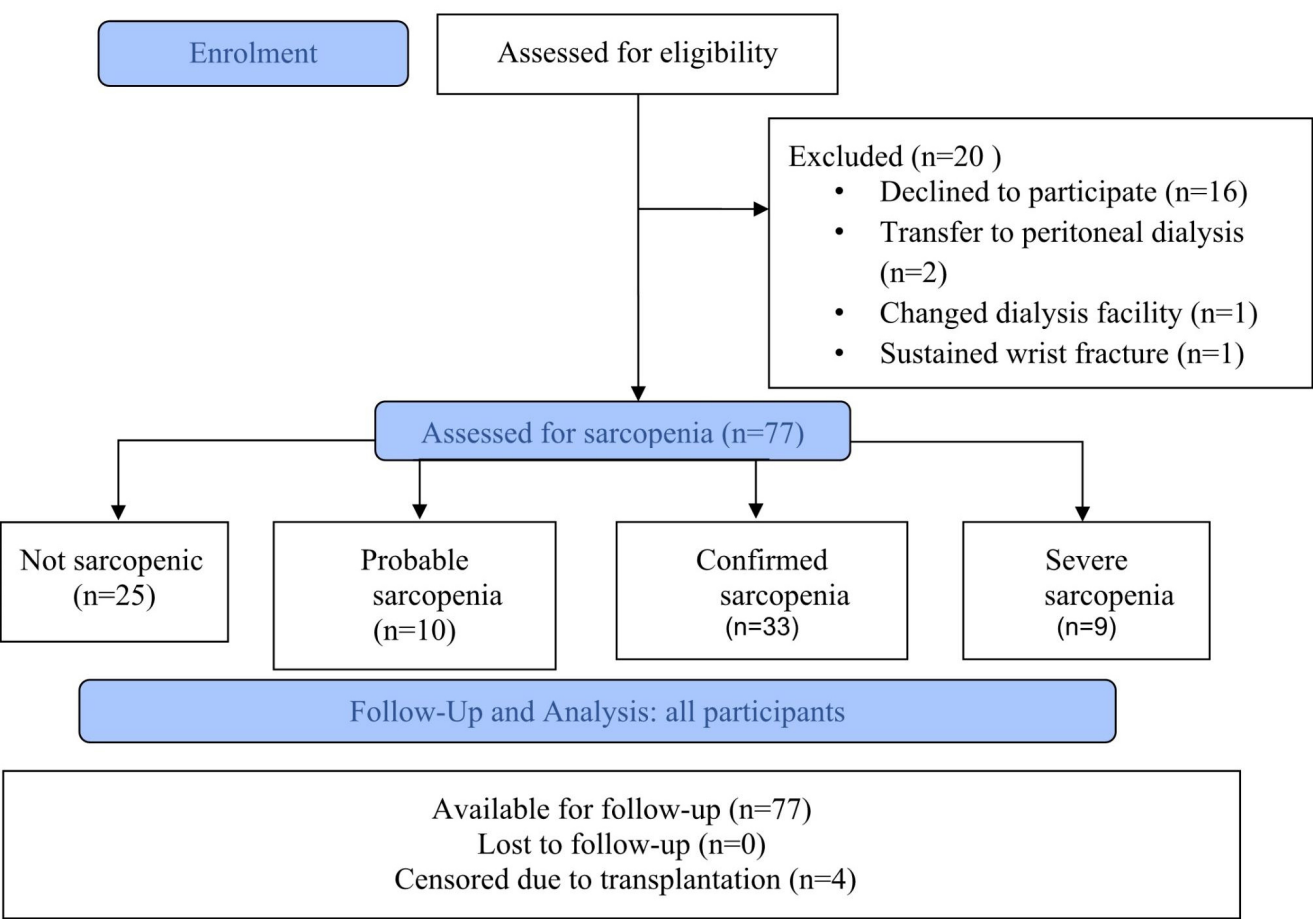
CONSORT Flow Diagram

Of 97 patients who met entry criteria, 16 declined participation and four did not undergo baseline testing (two transferred to peritoneal dialysis, one changed dialysis facility and one sustained wrist fractures precluding BIA) (Fig. 1).

Of the remaining 77 patients, 44 (57%) were male and the median age was 71 years (range 60–87 years). The majority (75%) were Caucasian, and the remainder were of Indian, Asian, Pacific Islander and other ethnicities. The aetiology of ESKD was diabetic nephropathy 40%, hypertension 32%, glomerulonephritis 10%, polycystic kidney disease 8%, other 16% and unknown 1%. Dialysis vintage, significant baseline comorbidities and biochemical data are included in Table 1. Mean values for urea reduction ratio (URR) > 70% and urea clearance by time and volume of distribution (Kt/V) $\ge$1.2 were consistent with adequate dialysis for all age categories, and biochemical measures were generally within the target range. BMI, and results for muscle strength, body composition, muscle mass and muscle function results are shown in Table 2.

**Table 1:** Baseline demographic and laboratory characteristics of study population

	Total	Males			Females		
Demographics and comorbidities	(n = 77)	60–69 (n = 19)	> 70 (n = 25)	Total (n = 44)	60–69 (n = 15)	> 70 (n = 18)	Total (n = 33)
Dialysis vintage (months)	40.9 (3-198)	35.2 (3-198)	41.6 (4-112)	37.8 (3-198)	33.5 (5–93)	51.8 (18–135)	45.1 (5-135)
Hypertension (%)	68 (88)	16 (84)	21 (84)	37 (84)	13 (87)	18 (100)	31 (94)
Ischaemic Heart Disease	37 (48)	16 (84)	13 (52)	29 (66)	4 (27)	4 (22)	8 (24)
Vascular disease*	25 (32)	4 (21)	14 (56)	18 (41)	2 (13)	5 (28)	7 (21)
Diabetes mellitus	39 (51)	13 (68)	11 (58)	24 (55)	10 (67)	5 (28)	15 (45)
Malignancy (ever)	20 (26)	2 (10)	10 (53)	12 (27)	3 (20)	5 (28)	8 (24)
Lung disease	14 (18)	3 (16)	7 (28)	10 (23)	3 (20)	1 (6)	4 (12)
Psychiatric diagnosis	20 (26)	5 (26)	7 (28)	12 (27)	4 (27)	4 (22)	8 (24)
Comorbidity score	2.9 ± 1.4	3.1 ± 1.4	3.3 ± 1.4	3.2 ± 1.4	2.6 ± 1.5	2.3 ± 1.2	2.5 ± 1.3
**Laboratory Data**							
Haemoglobin (g/L)	111.3 ± 13.2	110.1 ± 13.9	110.7 ± 13.3	110.4 ± 13.3	110.4 ± 13.1	114± 13.6	112.4 ± 13.1
Serum albumin (g/L)	37.6± 3.9	38.5± 2.5	37.3± 4.4	37.8± 3.7	38.5± 3.9	36.3± 4.3	37.3± 4.1
Calcium (mmol/L)	2.3 ± 0.2	2.3 ± 0.2	2.2 ± 0.1	2.3 ± 0.2	2.3 ± 0.2	2.3 ± 0.2	2.3 ± 0.2
Phosphate (mmol/L)	1.5 ± 0.4	1.6 ± 0.4	1.3 ± 0.2	1.4 ± 0.4	1.6 ± 0.4	1.4 ± 0.5	1.5 ± 0.4
Total cholesterol (mmol/L)	3.8 ± 0.9	3.7 ± 0.9	3.6 ± 0.8	3.7 ± 0.8	4.3 ± 1.2	3.9 ± 0.8	4.1 ± 1.0
Transferrin (g/L)	2.1 ± 1.2	2.5 ± 2.4	1.9 ± 0.5	2.2 ± 1.6	2.0 ± 0.3	1.9 ± 0.3	2.0 ± 0.3
CRP (mg/L)	13.3± 9.8	15.1± 12.7	12.3± 8.4	13.6± 10.0	8.3± 5.8	14.9± 11.2	12.7 ± 9.5
25(OH)D (nmol/L)	55.1± 9.8	53.7± 19.0	58.2± 21.4	56.2± 20.1	58.7± 39.8	49.4± 24.3	53.7± 31.6
iPTH (pmol/L)	30.5 (13.9, 58)	42.6 (12.4, 60)	29.5 (12.8, 53.9)	31.9 (12.9, 57.1)	27.1 (16.1,108.5)	30.4 (16.4, 67)	28 (16.8, 68.1)
URR (%)	76 ± 7	71 ± 4	74 ± 7	73 ± 6	80 ± 5	81 ± 3	80 ± 4
Kt/V	1.5 ± 0.3	1.2 ± 0.1	1.4 ± 0.2	1.3 ± 0.2	1.6 ± 0.2	1.7 ± 0.2	1.7 ± 0.2

Reported as median (interquartile range), number (%) or mean ± SD. Vascular disease* includes cerebrovascular accident, peripheral vascular disease, and abdominal aortic aneurysm. CRP: C-reactive protein; iPTH: intact-parathyroid hormone; URR: urea reduction ratio. Kt/V: dialysis adequacy: K clearance of urea, t dialysis time, V urea volume of distribution

**Table 2:** Baseline physical parameters

	Whole cohort (n = 77)	Male by age category			Female by age category		
		60–69 (n = 19)	> 70 (n = 25)	Total (n = 44)	60–69 (n = 15)	> 70(n = 18)	Total (n = 33)
Body mass index (kg/m^2^)	27.8 ± 6.0	30.1 ± 5.2	27.0 ± 5.0	28.3 ± 5.2	27.8 ± 8.7	26.3 ± 5.5	27.0 ± 6.9
Bioimpedence measures FTM (kg)	29.5 ± 12.1	32.3 ± 11.9	28.6 ± 11.9	30.2 ± 11.8	29.7 ± 16.2	27.8 ± 9.0	28.6 ± 12.4
FTI (kg/m^2^)	14.7 ± 6.3	15.0 ± 5.7	13.6 ± 5.5	14.2 ± 5.5	16.3 ± 9.5	14.6 ± 4.9	15.4 ± 7.2
LTM (kg)	33.6 ± 9.2	42.5±(9.7	34.8 ± 6.4	38.1 ± 8.7	27.2 ± 4.1	28.1 ± 7.3	27.6 ± 5.9
**LTI (kg/m** ^**2**^ **)**	12.1 ± 2.6	14.2 ± 2.7	12.2 ± 2.1	13.1 ± 2.5	10.8 ± 1.4	10.8 ± 2.6	10.8 ± 2.1
Muscle strength **Handgrip (kg)**	22.9 ± 8.7	29.4 ± 9.6	26.4 ± 6.9	27.7 ± 8.1	16.1 ± 4.7	16.8 ± 3.7	16.5 ± 4.1
Knee extension (kg) RCS (s)	22.4 ± 6.2 17.2 ± 7.2	26.1 ± 6.4 17.4 ± 9.4	24.1 ± 4.9 18.0 ± 6.5	25.0 ± 5.6 17.8 ± 7.7	18.9 ± 4.6 14.8 ± 5.8	19.2 ± 5.8 17.8 ± 6.8	19.1 ± 5.2 16.4 ± 6.4
Physical performance							
6MWT distance (m)	280.3± 114.9	317.9± 132.0	270.8± 132.3	291.1± 131.2	290.6± 91.9	245.3± 81.4	265.9± 86.6
**TUG (s)**	12.1 ± 5.5	10.9 ± 4.2	14.0 ± 7.3	12.6 ± 6.2	10.1±(2.5	12.5 ± 5.3	11.4 ± 4.3

Figures reported as mean ± SD. FTM: fat tissue mass; FTI: fat tissue index; LTM: lean tissue mass; LTI: lean tissue index; 6MWT: 6-minute walk test; TUG: timed-up-and-go; RCS: repeated chair stands. m: meters, s: seconds

Muscle mass as LTI was reduced in 79.5% of males and 57.6% of females. LTI was significantly associated with grip strength [Spearman’s rho (r_s_) 0.51, p < 0.001], knee extension strength (r_s_ 0.58, p < 0.001, RCS time (r_s_ − 0.37, p = 0.001), 6MWT distance (r_s_ 0.34, p = 0.003) and TUG (r_s_ − 0.25, p = 0.028). Grip strength and knee extension were positively correlated (r_s_ 0.49, p < 0.001), and RCS time was inversely related to knee extension strength (r_s_ − 0.35, p = 0.002), but RCS time was not significantly associated with grip strength. In the TUG, 15.9% of males and 6.1% of females took ≥ 20 s and in the 6MWT, 84.1% of males and 100% of females were unable to complete 400 m.

### Prevalence of sarcopenia

Grip strength is commonly used to differentiate patients without sarcopenia from those with probable sarcopenia, and grip strength and knee extension were positively correlated. Using grip strength, 32.5% of patients did not have sarcopenia, and probable sarcopenia was present in 13% of patients with reduced grip strength but normal LTI. Confirmed sarcopenia was present in 42.9% of patients with reduced grip strength and LTI. Because 91% of participants failed to reach 400 m in the 6MWT, the TUG was used to define severe sarcopenia, which was present in 11.7% of participants (Table 3).

**Table 3:** Presence of sarcopenia by chosen criteria and patient group

Patient Group Chosen criterion	No Sarcopenia (%) Normal grip strength	Probable Sarcopenia (%) Reduced grip strength	Confirmed Sarcopenia (%) Reduced grip strength + Reduced LTI	Severe Sarcopenia (%) Reduced grip strength + Reduced LTI + Reduced TUG
All (n = 77)	25 (32.5)	10 (13)	33 (42.9)	9 (11.7)
Male (n = 44)	13 (29.5)	4 (9.1)	20 (45.5)	7 (15.9)
Female (n = 33)	12 (36.4)	6 (18.2)	13 (39.4)	2 (6.1)
Age 60–69 years (n = 34)	18 (52.9)	5 (14.7)	10 (29.4)	1 (2.9)
Age > 70 years (n = 43)	13 (28.3)	4 (9.3)	18 (41.9)	8 (18.6)
Male 60–69 years (n = 19)	8 (42.1)	2 (10.5)	8 (42.1)	1 (5.3)
Male > 70years (n = 25)	5 (20)	2 (8)	12 (48)	6 (24)
Female 60–69 years (n = 15)	7 (46.7)	3 (2)	5 (33.3)	0 (0)
Female > 70 years (n = 18)	5 (27.8)	3 (16.7)	8 (44.4)	2 (11.1)

TUG: Timed up and go. LTI: lean tissue index measured by bioimpedance analysis

### Association between baseline characteristics and sarcopenia

Associations between baseline characteristics, and sarcopenia category are indicated in Table 4, and associations of sarcopenia with baseline biomarkers in Table 5. Compared to patients without sarcopenia, those with probable, confirmed and severe sarcopenia were older, had higher comorbidity scores and lower serum phosphate and transferrin values.

**Table 4:** Associations of baseline patient characteristics with sarcopenia

	Not Present N = 25	Probable N = 10	Confirmed N = 33	Severe N = 9	P-value
Dialysis Vintage (Months)	32 (15–70)	76.5 (36–136)	30 (19–55)	42 (32–81)	0.15
Female Sex	48% (12)	60% (6)	39% (13)	22% (2)	0.38
Age (years)	68 (63–72)	69 (65–79)	72 (66–74)	79 (73–81)	0.02
Comorbidity Score	3 (1–3)	2.5 (1–3)	3 (3–4)	4 (2–4)	0.04
BMI (kg/m^2^)	28.2 (24.2–30.1)	26.7 (23.2–30.8)	26.9 (23.5–30.9)	25.4 (21.8–29.8)	0.87
MAP (mmHg)	95 (87–108)	93 (78–98)	94 (87–105)	91 (83–101)	0.52
Substandard Nutrition	24% (6)	10% (1)	36% (12)	44% (4)	0.29

Substandard Nutrition = Nutritional assessment score rating B or C

Continuous variables summarised by medians and interquartile ranges. Differences assessed by the Kruskal-Wallis test. Categorical variables summarised by proportions and counts. Differences assessed by Fisher’s exact test

**Table 5:** Baseline biomarkers and their association with sarcopenia

	Not Present N = 25	Probable N = 10	Confirmed N = 33	Severe N = 9	P
Hb (g/L)	108 (99–116)	112 (111–128)	110 (101–119)	119 (113–121)	0.13
Albumin (g/L)	38 (36–41)	37.5 (35–40)	38 (35–40)	34 (33–40)	0.37
Ca (mmol/L)	2.25 (2.14–2.42)	2.34 (2.15–2.52)	2.28 (2.16–2.42)	2.21 (2.18–2.41)	0.81
PO_4_ (mmol/L)	1.55 (1.28–1.83)	1.74 (1.47–1.82)	1.32 (1.10–1.61)	1.20 (0.79–1.28)	0.01
25OHD (nmol/L)	54 (39–64)	51.5 (32–67)	46 (36–63)	55 (47–68)	0.85
Transferrin (g/L)	1.9 (1.8–2.2)	2.0 (1.8–2.1)	1.9 (1.7–2.2)	1.6 (1.4–1.6)	0.01
CRP (mg/L)	4.5 (1–13)	2.5 (1–7)	5 (1–13.5)	11 (4–17)	0.41
PTH (pmol/L)	31.3 (14.1–82.1)	31.2 (11.5–66.5)	29.8 (15.7–48.6)	22.4 (13.4–60.1)	0.95

Results are expressed as median and interquartile ranges, with Kruskal-Wallis tests for each biomarker

In post hoc pairwise comparisons with Bonferroni adjustment, patients with severe sarcopenia were older than those without sarcopenia (p = 0.020), patients with severe sarcopenia had lower serum phosphate values than those with no or probable sarcopenia (p = 0.032 and p = 0.022 respectively) and patients with severe sarcopenia had lower serum transferrin values than those with no or confirmed sarcopenia (p = 0.010 and p = 0.009 respectively).

### Outcomes

At six years follow up, 65% (n = 50) of patients had died, 38% due to CV events, 30% through withdrawal from treatment, 14% from infection and 18% from other causes. Reasons for withdrawal from treatment included dementia (n = 1), palliation (n = 1), malignancy (n = 2) and psychosocial reasons (n = 6). Four patients were censored due to transplantation.

**Fig. 2:**
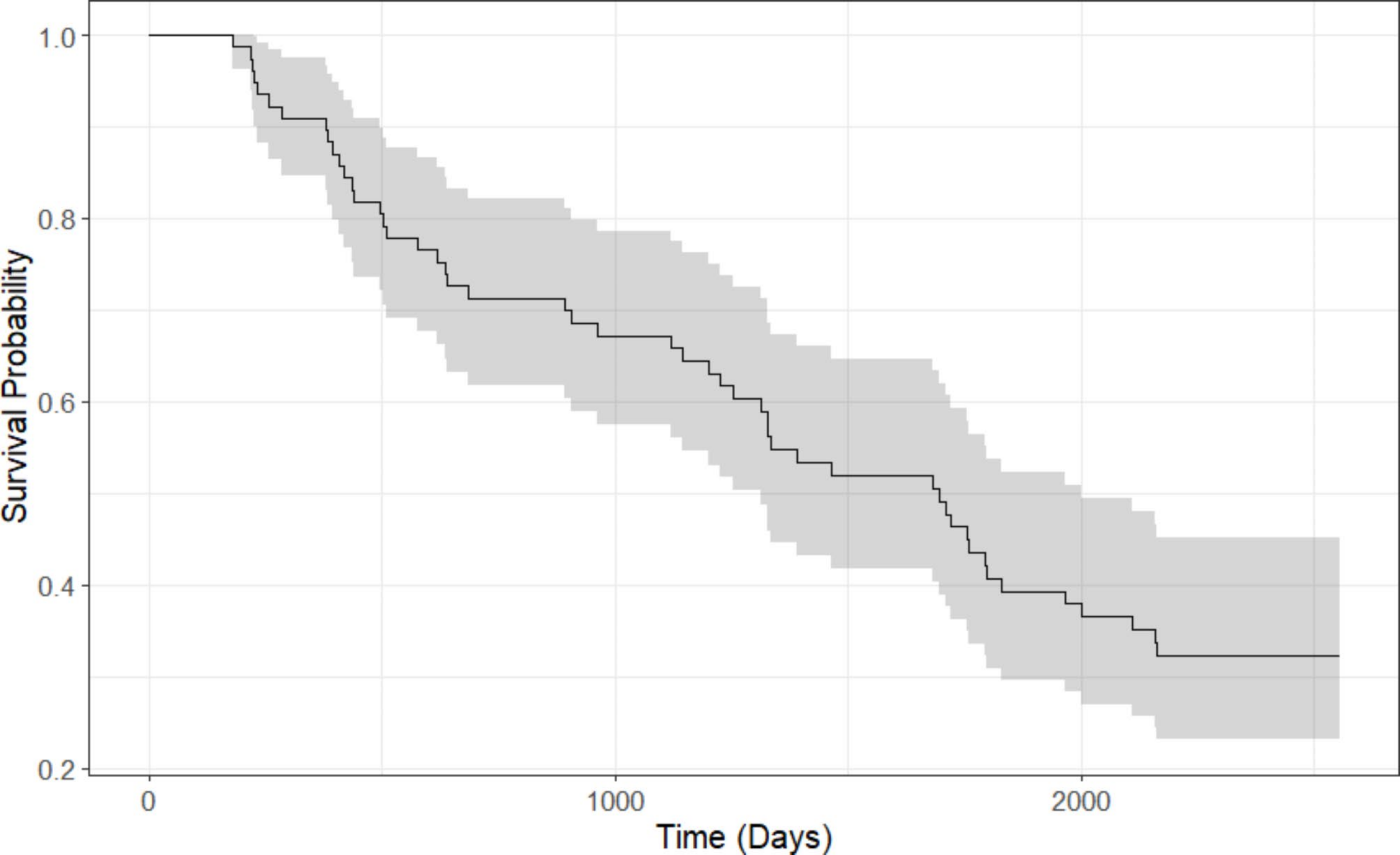
Probability of survival from study inclusion (n = 77)

For patients who died, the median time from study commencement till death was 2.8 years (1.2, 4.7), with a median time on dialysis prior to the study of 2.8 years (1.3, 4.8). For patients who were alive and censored at the end of follow up, their time on dialysis from study commencement was 6 years, and median time on dialysis prior to the study was 3.1 years (1.7, 6.8). Survival probability is shown in Fig. 2.

Using Kaplan-Meier survival analysis, median survival in patients without sarcopenia was 59.9 months (95% CI: 30.3, 89.6), those with probable sarcopenia 56 months (95% CI: 42.8, 69.2), confirmed sarcopenia 58.7 months (95% CI: 37, 80.3) and severe sarcopenia 21.3 months (95% CI: 1.8, 40.7). A log-rank test comparing the survival distribution between groups showed no significant differences: χ^2^(3) = 5.827, *p* = 0.120. For pairwise comparison of patients without sarcopenia versus severe sarcopenia, p = 0.045 was non-significant after Bonferroni adjustment.

Baseline patient characteristics likely to influence survival were next assessed over time by univariate proportional hazards regression (Table 6).

**Table 6:** Unadjusted hazard ratio of baseline characteristic

Characteristic	Hazard Ratio	95% Confidence Interval	P
Sarcopenia Absent			
Probable	1.24	0.52, 2.97	0.62
Confirmed	0.96	0.49, 1.91	0.91
Severe	2.46	1.03, 5.90	0.04
Dialysis Vintage (Months)	1.001	1.00, 1.01	0.75
Female Sex	0.86	0.49, 1.50	0.59
Age (years)	1.02	0.97, 1.06	0.49
Comorbidity Score	1.21	0.99, 1.47	0.06
BMI (kg/m^2^)	1.02	0.97, 1.07	0.44
MAP (mmHg)	0.97	0.95, 0.99	< 0.01
Substandard Nutrition	1.13	0.62, 2.05	0.69

Substandard Nutrition = Nutritional assessment score rating B or C.  MAP: mean arterial pressure (median (range)) 94 (69,131) mmHg. Comorbidity Score (mean±SD) 3.0±1.4.

Each characteristic has undergone a univariate proportional hazards regression

Serum transferrin and phosphate values were not associated with mortality in this analysis

**Fig. 3:**
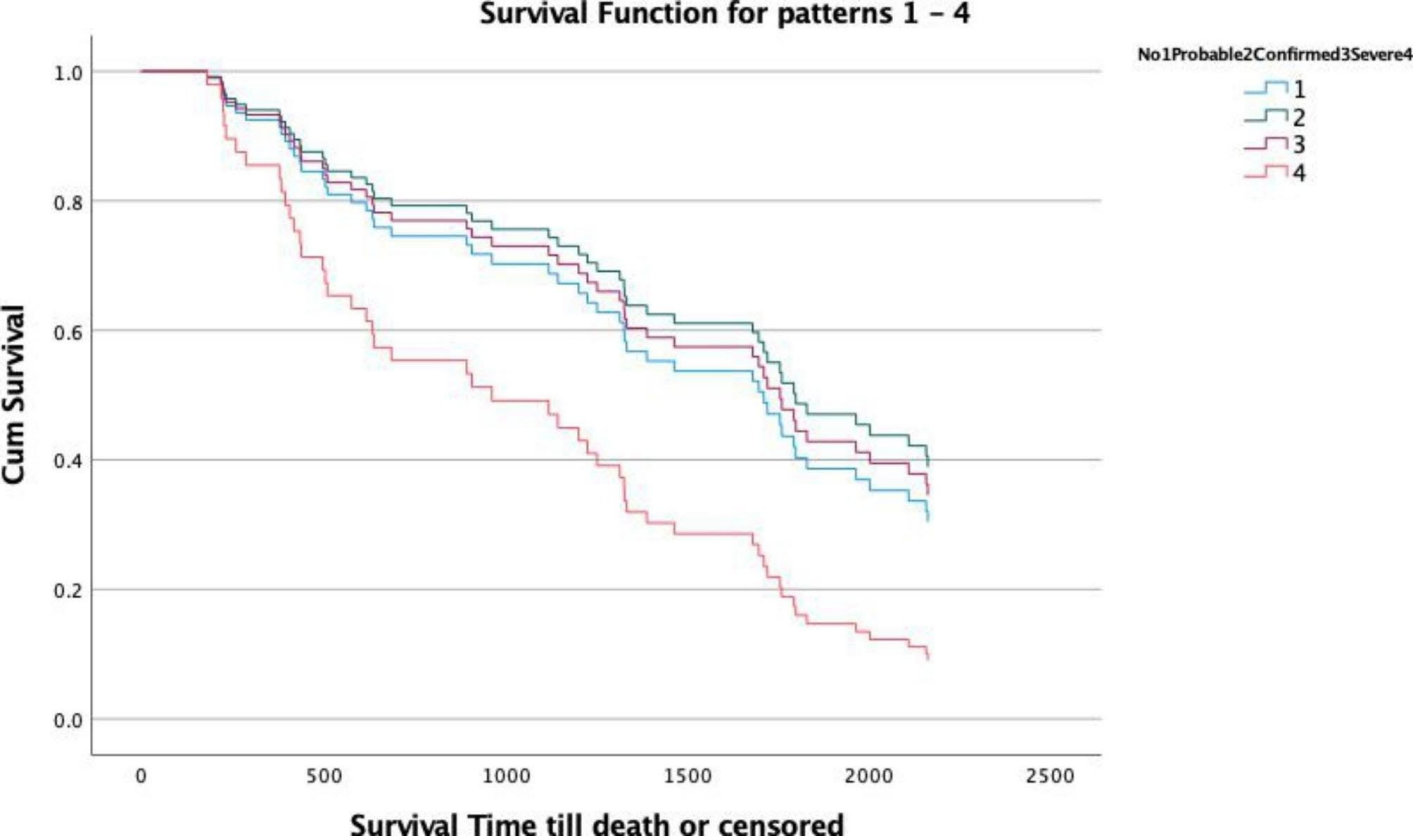
Cox proportional hazard analyses, showing survival curves for patients with absent, probable, confirmed and severe sarcopenia, adjusted for age, dialysis vintage, mean arterial pressure and the total comorbidity score

Patient characteristics that differed between sarcopenia grades, were significant in the univariate proportional hazard regression, or were clinically plausible, were entered into a Cox proportional hazards model. The final model included sarcopenia (absent, presumed, confirmed or severe), age, dialysis vintage, mean arterial pressure and the total comorbidity score (Fig. 3).

In the model, higher baseline comorbidity scores and lower MAP predicted mortality (Table 7).

**Table 7:** Characteristics entered into the Cox proportional hazards model

Characteristic	Hazard Ratio	95% Confidence Interval	P-value
Sarcopenia Absent			
Probable	0.79	0.31, 2.01	0.62
Confirmed	0.89	0.44, 1.84	0.76
Severe	2.02	0.78, 5.23	0.15
Dialysis Vintage (Months)	1.003	1.00, 1.01	0.44
Age (years)	0.99	0.94, 1.04	0.60
Comorbidity Score	1.27	1.02, 1.58	0.03
MAP (mmHg)	0.96	0.94, 0.99	< 0.01

Adding the nutritional assessment score or values of serum transferrin or phosphate

did not improve the model. In a Cox regression with censoring for death due to cardiovascular disease and excluding death due to withdrawal from dialysis for psychosocial reasons, infection, or other non-cardiovascular causes, sarcopenia status was not associated with cardiovascular mortality. The total comorbidity score (HR 1.57, p = 0.02)) and the baseline MAP (HR 0.96, p = 0.03) remained significant. Low grip strength was present in 40.3% of patients, but 50.6% took > 15 s for 5 repetitions of the RCS. If sarcopenia was diagnosed by abnormal RCS and / or grip strength, 25 patients (32.5%) did not have sarcopenia while 52 patients (67.5%) could be classified with sarcopenia (probable, confirmed or severe). Replacing sarcopenia classified using grip strength with sarcopenia classified by RCS and / or grip strength, plus LTI and TUG in the Cox analysis, associations with mortality remained for the total comorbidity score and MAP, but sarcopenia was not associated with the outcome (Supplementary Table 1). When patients were assessed as having severe sarcopenia (n = 9; 11.7%) versus other categories, the total comorbidly score and MAP remained significant, while the presence of severe sarcopenia was of borderline significance (HR 2.22; 95% CI 0.99, 5.01, p = 0.054) (Supplementary Table 2). When sarcopenia was substituted in the Cox analyses by either reduced grip strength, reduced LTI or increased TUG, none independently predicted mortality (Supplementary Tables 3a, 3b and 3c), whereas MAP and the total comorbidity score remained significant.

## Discussion

### Prevalence of sarcopenia in patients on haemodialysis

In this study probable, confirmed or severe sarcopenia was present in 67.6% of elderly, stable satellite dialysis patients, with a median age of 71 years, using EWGSOP2 criteria for diagnosis. In a 2014 study of adults receiving haemodialysis, the prevalence of sarcopenia depended on the criteria and assessment method used [20]. Confirmed sarcopenia estimated from DXA appendicular lean mass index (ALMI) and grip strength ranged from 31 to 63% depending on ALMI cut points [20]. Using BIA and grip strength, confirmed sarcopenia ranged from 13 to 45%, depending on lean body mass index BIA cutpoints [20].

Patients on dialysis have a high risk for developing sarcopenia due to anorexia, poor nutrient intake, dialysis related factors such as nutrient loss into dialysate fluid, acidosis, chronic inflammation, comorbid illnesses and hormonal disorders [21]. While in the general population, sarcopenia is associated with frailty, functional impairment, disability, reduced quality of life and mortality, in dialysis populations its ability to predict mortality is not well described [9, 22]. In this study, mortality was not predicted in a number of models by the absence or presence of sarcopenia, although severe sarcopenia was likely to influence survival risk in univariate analysis [HR 2.46 (95% CI 1.03–5.90)] (Table 6), and the influence of severe sarcopenia may have been limited by patient numbers. On the other hand, lower baseline MAP and a higher total comorbidity score (which including a number of cardiovascular risk factors) predicted all-cause and cardiovascular mortality in adjusted analyses. This is not surprising, because cardiovascular disease is the leading cause of death in dialysis patients, and lower blood pressure may signify cardiovascular pathology. No other assessed baseline variables were independently associated with mortality in Cox analyses. The phenomenon of traditional risk factors performing poorly in patients with CKD is well recognised, and likely to be caused by the high number of competing risks these vulnerable patients have for mortality [23]. Information on educational level, smoking, alcohol intake and physical activity was not collected at baseline. However, end organ damage resulting from lifestyle factors is reflected in the comorbidity index, which included hypertension, ischaemic heart disease, other vascular disease, respiratory disease, malignancy, and diabetes. Only 16% of males and no females were able to complete 400 m in the 6-minute walk test, indicating that this group had limited exercise capacity prior to study entry.

### Assessing prognosis in patients on haemodialysis

Several prognostic markers have been recommended for use in patients with CKD. In an earlier study of patients on haemodialysis, those with high BMI and normal or high muscle mass (based on 24-hour urinary creatinine excretion) had a lower hazard ratio for death than patients with a normal BMI [24], however patients with high BMI and low muscle mass did not have improved survival. Similarly, improved survival of maintenance haemodialysis patients has been associated with greater mid-arm muscle circumference, which is a surrogate for lean body mass [25]. On the other hand, another study of haemodialysis patients reported that high fat mass provided a survival advantage in both sexes, whereas a higher lean body mass was only protective in women, [26] and a study of patients commencing dialysis reported a survival advantage for patients with a BMI > 25 kg/m^2^ and those with higher fat body mass index [27].

Irrespective of BMI, survival is reported to be lower for patients defined as having PEW based on assessment by SGA, and the SGA has been associated with increased mortality in other studies, including over 7 years for patients with a mean age of 59 years on haemodialysis [28] and over 4 years for patients on peritoneal dialysis [29]. A number of modifications of the SGA have been used in patients on dialysis, including a ‘dialysis malnutrition score,’ which was reported to correlate with biochemical parameters associated with malnutrition more closely than the SGA [30]. Nevertheless, the lack of uniformity between versions of the SGA does make it difficult to compare results for nutritional status between studies, and to provide consistent methodology guidance to clinicians [31]. The subjective nutritional assessment score used in this study was adapted from the SGA, but excluded functional capacity, comorbidities, and body composition, because these were quantitated by methods less prone to subjective error.

Other indices found to predict early mortality following commencement of dialysis include age, comorbidities, and recent hospitalisation, but most of these perform only moderately well, and more accurate tools are required [32]. Three-year mortality was predicted in incident dialysis patients using patient demographics, comorbid conditions and laboratory variables with a C statistic of 0.73 (95% CI, 0.71–0.76) [33] and a ‘new comorbidity index’ assigning weights to each of 11 comorbidities has shown good predictive value in older dialysis patients followed for nearly 10 years [34]. However, no model has been accepted into general use [7].

An important question is why our results differ from those of some reported studies showing positive associations between sarcopenia and mortality in patients on dialysis. In a recent meta-analysis, [7] sarcopenia (defined as low muscle mass plus low muscle strength or performance) was reported to predict mortality in 8 studies that included dialysis patients, although four of these were non-significant after adjustment for covariates. The mean age of participants in 3 of the 4 positive studies was $\le$61 years, which is younger than the mean age of our cohort, and known cardiovascular disease was not included as a covariate in all positive studies. Two positive studies had fewer patients than the current study. Selection bias may have contributed to our outcome, because not all patients were suitable to undergo BIA or baseline functional testing. However, particularly for older patients, choices to commence dialysis or to proceed down a non-dialysis, supportive care pathway, are generally based on discussion between the patient, family, medical and allied health team members, focussing on the benefits of dialysis to quality of life and survival. Older dialysis patients have therefore undergone extensive filtering, and are likely to be healthier, with a better prognosis for survival, than patients offered management through a non-dialysis, renal supportive care program. For patients in this study, the relatively long period from commencing dialysis until death (median 5.6 years) or end of follow up (median 9.1 years) may reflect such selection bias. If all prospective patients were commenced on dialysis without allocation bias, sarcopenia might have impacted mortality differently. Selection may have even greater impacts as more elderly patients are considered for dialysis. Selection bias may also have influenced our finding that the nutritional assessment score, which excluded independently assessed physical performance, body composition and comorbidities, did not predict mortality. In addition, the nutritional assessment score was not associated with the absence of sarcopenia, or category of sarcopenia at baseline, although lower serum phosphate and lower transferrin, which reflect nutritional status, were associated with baseline sarcopenia. However, neither improved prediction of mortality in Cox models. On the other hand, a lower MAP, and the total comorbidity score, which can be determined from patient records or a simple questionnaire, were predictive.

### Defining sarcopenia

A variety of diagnostic criteria have been used to define sarcopenia, resulting in inconsistent estimates of its prevalence and impact. However, use of the EWGSOP2 criteria has improved the ability of clinicians to establish a diagnosis. The current focus of these criteria on muscle strength, then muscle quantity or quality and finally performance, represents a shift from earlier definitions based primarily on measurement of appendicular or skeletal muscle mass, or LTI (kg/m^2^). Recommended cut-points are generally 2 to 2.5 SD below mean reference values derived from meta-analysis of studies recruiting healthy young adults.

Muscle strength can be assessed by several validated techniques, and this study utilised grip strength, while also assessing knee extension and recurrent chair stands. Grip strength is simple and inexpensive, and in the general population is independently associated with poor patient outcomes, including prolonged hospitalization, functional limitations, reduced quality of life and mortality [6]. However, in this study mortality was not predicted when substituting the sarcopenia category with low grip strength.

Whilst MRI and CT are considered the gold standard for measuring muscle mass [6], and DXA is a recommended reference method, BIA is widely accessible, portable, affordable, easy to use, has no radiation and has been validated against other techniques [35]. In addition, BIA is available in many dialysis units, where it is used to assist the evaluation of fluid status. Because estimates of muscle mass differ between BIA machines and reference populations, the EWGSOP2 suggests that raw measures are preferable [6]. This study classified sarcopenic-muscle mass as $\ge$2 SD from a gender-matched young reference range. Using that definition, 79.5% of male and 57.6% of female patients could be classified to have a LTI in the sarcopenic range. However, in this study, the LTI was not significantly associated with mortality. This is consistent with a study of 330 incident dialysis patients, with 23% aged >65 years, of whom 20% fulfilled criteria for confirmed sarcopenia [9]. Over a median follow-up of 29 months, low muscle mass alone was not associated with increased mortality, whereas individuals with low muscle strength had increased mortality, irrespective of their muscle mass.

To assess muscle function (physical performance), we used the validated TUG and 6MWT. By EWGSOP2 criteria, and with TUG as the physical performance measure, probable, confirmed, or severe sarcopenia was present in 67.6% of patients in this study, with 70.5% of men and 63.6% of women fulfilling criteria for sarcopenia. TUG did not predict mortality when normal / increased TUG was substituted for sarcopenia category in the Cox analysis.

### Limitations

Strengths of this study include use of the ANZDATA registry, which contains information on all patients receiving renal replacement therapy in Australia, resulting in complete follow-up. We also used robust, validated methods to test muscle strength and function and applied current sarcopenia definitions. Limitations include the observational nature of the study design, potential for residual confounders and a relatively small sample size. Nevertheless, we are not aware of other studies that have assessed the survival of patients on haemodialysis using the 2019 EWGSOP2 sarcopenia criteria, and despite the relatively small numbers, mortality was 65% at 6 years. There may also be demographic differences between our participants and other patients on dialysis that limit the generalizability of our results, because Caucasians made up 75% of participants, and Caucasian reference values were used. Fluid retention can influence body composition calculations using BIA; however, we minimized that risk by assessing body composition following a mid-week dialysis session with patients at their ‘dry weight’. There was also potential for exclusion bias, by excluding patients unable to complete baseline functional testing.

## Conclusion

Probable, confirmed, or severe CKD-related sarcopenia was present in approximately two-thirds of elderly haemodialysis patients in this cohort, with a mortality of 65% over 6 years follow-up. In adjusted models, lower MAP and a higher total comorbidity score were significantly associated with an increased risk of mortality, whereas the presence or category of sarcopenia, baseline laboratory indices and the subjective nutritional assessment score were not associated. These findings suggest that risk factors other than sarcopenia determine the prognosis of older patients accepted onto dialysis when a renal supportive care program is an option, and the prognosis of patients who continue on dialysis into older age. Whilst identification of sarcopenia through assessment of muscle strength, muscle mass and physical performance is predictive of mortality in the general population and in some studies of patients on dialysis [7], it was of limited value in older dialysis patients participating in this study. However, both a lower MAP and higher total comorbidity score, which is readily calculated from patient data, were predictive of mortality. If confirmed in other similarly aged dialysis cohorts, both could contribute to discussions regarding prognosis between health professional, and elderly patients on dialysis and their families.

## Electronic supplementary material

Below is the link to the electronic supplementary material.

Supplementary Material 1

## Data Availability

Deidentified data is available on request from Dr Grahame Elder.
