# Supplementary material for: Chronic kidney disease-related sarcopenia as a prognostic indicator in elderly haemodialysis patients
Source: BMC Nephrol. 2023 May 19;24:138. doi: 10.1186/s12882-023-03175-5 (PMC10199600; doi:10.1186/s12882-023-03175-5)
Supplement: Supplementary file 1 — Supplementary Material 1 [file 12882_2023_3175_MOESM1_ESM.docx]

Supplementary Table 1. Replacing sarcopenia classified using grip strength with sarcopenia classified by recurrent chair stands and / or grip strength in the Cox analysis. MAP: mean arterial pressure.

| **Variables in the Equation** | | | | | | | | |
| --- | --- | --- | --- | --- | --- | --- | --- | --- |
|  | B | SE | Wald | df | Sig. | Exp(B) | 95.0% CI for Exp(B) | |
|  |  |  |  |  |  |  | Lower | Upper |
| Age (years) | -.013 | .025 | .284 | 1 | .594 | .987 | .940 | 1.036 |
| Dialysis Vintage (months) | .003 | .003 | .632 | 1 | .427 | 1.003 | .996 | 1.009 |
| Total comorbidity score | .240 | .112 | 4.613 | 1 | .032 | 1.271 | 1.021 | 1.583 |
| MAP (mm Hg) | -.038 | .013 | 9.409 | 1 | .002 | .962 | .939 | .986 |
| Sarcopenia (1-4) based on Grip strength and / or recurrent chair stands |  |  | 3.930 | 3 | .269 |  |  |  |
| Sarcopenia (1) | -.233 | .477 | .238 | 1 | .626 | .793 | .311 | 2.019 |
| Sarcopenia (2) | -.114 | .367 | .097 | 1 | .755 | .892 | .435 | 1.830 |
| Sarcopenia (3) | .701 | .486 | 2.082 | 1 | .149 | 2.015 | .778 | 5.218 |

Supplementary Table 2. Cox analysis for severe sarcopenia versus all other categories. MAP: mean arterial pressure.

| **Variables in the Equation** | | | | | | | | |
| --- | --- | --- | --- | --- | --- | --- | --- | --- |
|  | B | SE | Wald | df | Sig. | Exp(B) | 95.0% CI for Exp(B) | |
|  |  |  |  |  |  |  | Lower | Upper |
| Age (years) | -.013 | .024 | .293 | 1 | .588 | .987 | .941 | 1.035 |
| Dialysis Vintage (months) | .002 | .003 | .529 | 1 | .467 | 1.002 | .996 | 1.009 |
| Total comorbidity score | .237 | .109 | 4.751 | 1 | .029 | 1.267 | 1.024 | 1.568 |
| MAP (mm Hg) | -.037 | .012 | 10.387 | 1 | .001 | .963 | .942 | .986 |
| Severe Sarcopenia by TUG | -.798 | .415 | 3.698 | 1 | .054 | .450 | .200 | 1.015 |

Supplementary Table 3a: Substitution of grip strength for sarcopenia category in the Cox model. MAP: mean arterial pressure.

| **Variables in the Equation** | | | | | | | | |
| --- | --- | --- | --- | --- | --- | --- | --- | --- |
|  | B | SE | Wald | df | Sig. | Exp(B) | 95.0% CI for Exp(B) | |
|  |  |  |  |  |  |  | Lower | Upper |
| Age (years) | -.006 | .024 | .071 | 1 | .790 | .994 | .949 | 1.041 |
| Dialysis Vintage (months) | .002 | .003 | .275 | 1 | .600 | 1.002 | .995 | 1.008 |
| Total comorbidity score | .241 | .108 | 4.926 | 1 | .026 | 1.272 | 1.029 | 1.573 |
| MAP (mm Hg) | -.036 | .011 | 10.345 | 1 | .001 | .964 | .943 | .986 |
| Grip strength (<27 kgF males and <16 kgF females) | -.220 | .301 | .534 | 1 | .465 | .802 | .444 | 1.448 |

Supplementary Table 3b: substitution of reduced lean tissue index (LTI) below the tenth centile for sarcopenia category in the Cox model. MAP: mean arterial pressure.

| **Variables in the Equation** | | | | | | | | | |
| --- | --- | --- | --- | --- | --- | --- | --- | --- | --- |
|  | B | SE | Wald | df | Sig. | Exp(B) | 95.0% CI for Exp(B) | |  |
|  |  |  |  |  |  |  | Lower | Upper |  |
| Age (years) | .000 | .023 | .000 | 1 | .983 | 1.000 | .957 | 1.046 |  |
| Dialysis Vintage (months) | .002 | .003 | .519 | 1 | .471 | 1.002 | .996 | 1.009 |  |
| Total comorbidity score | .270 | .107 | 6.314 | 1 | .012 | 1.309 | 1.061 | 1.616 |  |
| MAP (mm Hg) | -.036 | .011 | 10.118 | 1 | .001 | .965 | .944 | .986 |  |
| LTI below tenth centile | -.365 | .290 | 1.581 | 1 | .209 | .694 | .393 | 1.226 |  |

| Supplementary Table 3c: substitution of increased timed up and go (TUG) for sarcopenia category in the Cox model. MAP: mean arterial pressure.  **Variables in the Equation** | | | | | | | | |
| --- | --- | --- | --- | --- | --- | --- | --- | --- |
|  | B | SE | Wald | df | Sig. | Exp(B) | 95.0% CI for Exp(B) | |
|  |  |  |  |  |  |  | Lower | Upper |
| Age (years) | -.013 | .024 | .293 | 1 | .588 | .987 | .941 | 1.035 |
| Dialysis vintage (months) | .002 | .003 | .529 | 1 | .467 | 1.002 | .996 | 1.009 |
| Total comorbidity score | .237 | .109 | 4.751 | 1 | .029 | 1.267 | 1.024 | 1.568 |
| MAP (mm Hg) | -.037 | .012 | 10.387 | 1 | .001 | .963 | .942 | .986 |
| Increased TUG | -.798 | .415 | 3.698 | 1 | .054 | .450 | .200 | 1.015 |
